# Durable vesicles for reconstitution of membrane proteins in biotechnology

**DOI:** 10.1042/BST20160019

**Published:** 2017-02-15

**Authors:** Paul A. Beales, Sanobar Khan, Stephen P. Muench, Lars J.C. Jeuken

**Affiliations:** 1 School of Chemistry and Astbury Centre for Structural Molecular Biology, University of Leeds, Leeds LS2 9JT, U.K.; 2 School of Biomedical Sciences and Astbury Centre for Structural Molecular Biology, University of Leeds, Leeds LS2 9JT, U.K.

**Keywords:** bionanotechnology, block copolymers, *in vitro* reconstitution, membrane proteins, synthetic biology, vesicles

## Abstract

The application of membrane proteins in biotechnology requires robust, durable reconstitution systems that enhance their stability and support their functionality in a range of working environments. Vesicular architectures are highly desirable to provide the compartmentalisation to utilise the functional transmembrane transport and signalling properties of membrane proteins. Proteoliposomes provide a native-like membrane environment to support membrane protein function, but can lack the required chemical and physical stability. Amphiphilic block copolymers can also self-assemble into polymersomes: tough vesicles with improved stability compared with liposomes. This review discusses the reconstitution of membrane proteins into polymersomes and the more recent development of hybrid vesicles, which blend the robust nature of block copolymers with the biofunctionality of lipids. These novel synthetic vesicles hold great promise for enabling membrane proteins within biotechnologies by supporting their enhanced *in vitro* performance and could also contribute to fundamental biochemical and biophysical research by improving the stability of membrane proteins that are challenging to work with.

## Introduction

The significant challenge of maintaining membrane proteins in their native state, preserving their structure and function *in vitro*, calls for experimental tools that facilitate their study and handling, which are being continually developed [1,2]. These efforts are driven by their abundance, comprising approximately one-third of the proteome, and their importance in biological function, as pharmaceutical targets [3] and their future potential within emerging nano- and bio-technologies [4,5].

Integral membrane proteins (IMPs) span the lipid bilayers that form functional barriers at the interface of cellular and subcellular compartments. They perform diverse roles such as adhesion, material transport, signal transduction and catalysis. Besides their well-established importance in drug-screening programmes, IMPs are of interest as biofunctional components within technologies, including sensors, nanoreactors, protocells and nanomedical drug delivery systems [6–8]. The construction of such novel artificial biological devices comprises the field of Synthetic Biology [9,10], which is a major growth area in current fundamental and applied research.

The biggest hurdle to overcome for manipulation of IMPs *ex vivo* is their inherent instability in water. In their native state, a large proportion of the exterior surface of IMPs is hydrophobic to enable their stable insertion into a biomembrane. These non-polar surface residues must be shielded from direct contact with water, as is achieved by the liquid crystalline amphiphilic solvation within a lipid bilayer. A wide variety of self-assembling and macromolecular materials have been applied to IMP handling; these soft materials combine both liquid-like and solid-like properties, known as mesophases, and can mimic important aspects of their native environment [11]. The most commonly used IMP-stabilising systems are detergent micelles, but other materials have been developed for enhanced stability, more native-like environments or practical simplicity: these include amphiphilic polymers called amphipols [12], and disc-shaped lipid-containing structures such as bicelles [13], lipoprotein-mimetic nanodiscs [14], saposin lipoprotein nanoparticles [15] and styrene maleic acid lipid particles (SMALPs) [16]. Structurally complex bicontinuous cubic phases of surfactants are also used in crystallisation studies [17]. These soft, self-organised systems have facilitated important advances in our understanding of the structure and function of membrane proteins.

Despite the variety and success of the aforementioned materials, an important feature of natural organisation is lost: compartmentalisation [18,19]. Many IMPs transport ions or molecules from one distinct aqueous compartment to another or are driven by the release of energy from transmembrane potential differences. Moreover, the loss of a ‘closed’ system is problematic for structural studies where the detergent-extracted IMP may not exist in its native state due to the lack of a membrane potential. Compartments can be achieved by reconstitution into liposomes with the added benefit of the native-like lipid bilayer structure. More complex droplet interface bilayer systems can also provide compartmentalised architectures [20,21]. Liposome reconstitution protocols have widely been reported [22], yet novel materials and methods are still continually being developed [23,24].

While liposomes solve the challenges of compartments and native-like solvation, their major drawback is the lack of long-term stability of these systems [25,26]. This is particularly problematic for biotechnological applications, where considerable stability and durability are necessities. Alternative soft matter systems are known to form tougher vesicles than liposomes and so have become candidate constructs for IMP stabilisation: polymersomes.

## Polymersomes

**Figure 1.**
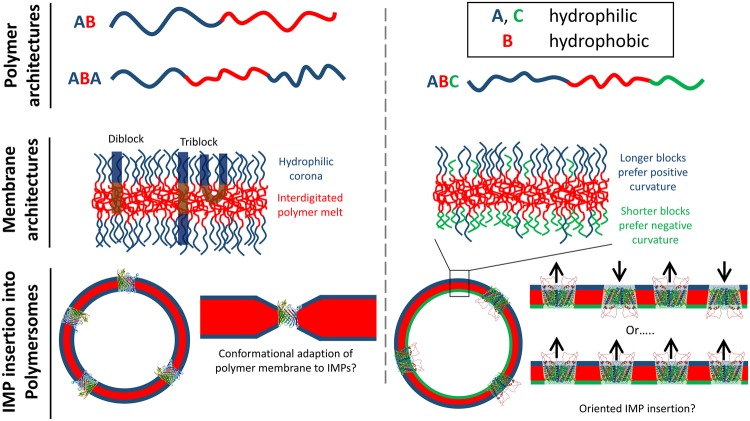
Polymersomes. Polymer membranes are formed from amphiphilic block copolymers that often have AB, ABA or ABC polymer architectures. These membranes are interdigitated with a viscous polymer melt at its core and a hydrophilic corona of polymers in an extended brush-like conformation. Triblock copolymers may be a mixture of transmembrane and hairpins, which have both their hydrophilic blocks displayed at the same membrane surface. AB and ABA architectures and resulting polymerosome structures are shown on the left-hand side. Asymmetric ABC polymers (right-hand side) can give rise to asymmetric membrane chemistries if the hydrophilic block lengths are different: longer polymers prefer the positive curvature at the exterior of the vesicle and vice versa. Membrane proteins can be inserted into these polymer membranes even if the polymer membrane is much thicker than the hydrophobic thickness of the IMP, suggesting conformational adaption of the polymers to the protein, shown for OmpF (PDB ID: 2OMF) on the left-hand side [30]. Asymmetric ABC membranes may also help drive preferential orientation of the IMPs within the membrane, shown for Aqp0 (PDB ID: 2B6P) on the right-hand side [31].

Polymersomes are composed of amphiphilic polymers that spontaneously self-assemble in water to form vesicles, analogous to the formation of liposomes from their constituent lipids [27]. While there are many commonalities between liposomes and polymersomes, there are also many important fundamental differences. The advantages of polymer membranes over their lipid counterparts are their broader parameter space of physical and chemical properties due to the variety of polymer chemistries that can be applied and their broad range of possible molecular masses. Amphiphilic block copolymers that are known to self-assemble into vesicles can have several architectures, the most common being diblock AB copolymers (A = hydrophophilic polymer, B = hydrophobic polymer), or triblock ABA or ABC copolymers, where A and C are chemically distinct hydrophilic blocks (Figure 1). The relative block lengths required for vesicle assembly can be correlated with packing parameter models for amphiphile self-assembly [28], for example worm-like micelles commonly assemble if the hydrophilic to hydrophobic block volume ratios are slightly too large for vesicles to be preferred [29].

Unlike lipid membranes, block copolymer membranes do not form a distinct bilayer structure, i.e. two molecular monolayers aligned in apposition to one another. Instead the hydrophobic polymer blocks interdigitate and penetrate at random within the hydrophobic core of the membrane in an entangled polymer melt [32]. Polymersome membranes have been reported ranging in thickness from that of a lipid membrane (3–5 nm) up to 40 nm [33]. Polymersomes generally have a membrane permeability that is lower than those of lipid membranes due to the inverse dependence of permeability on membrane thickness [34]. The membrane fluidity of viscous polymer membranes is also typically at least an order of magnitude less than for lipid bilayers, and the mechanical properties of tough, robust polymer membranes can range over at least three orders of magnitude [35].

Common hydrophobic blocks include polybutadiene (PBd), polystyrene (PS) and polydimethylsiloxane (PDMS), while common hydrophilic blocks are poly(ethylene oxide) (PEO), poly(acrylic acid) (PAA) and poly(2-methyl oxazoline) (PMOXA). Despite their vastly different chemistry, structure, mechanics and dynamics, polymersomes have proved successful reconstitution systems for some IMPs. Key to this success are the flexibility of the polymer chains and the hydrophobic thickness of the membrane: flexible, linear hydrophobic polymers allow conformational adaption to the preferred hydrophobic thickness of the protein (Figure 1) and, while membrane thickness may in some cases be less critical, membranes closer to the natural hydrophobic thickness of a biomembrane can be preferable. While not IMPs *per se*, gramicidin and ionomycin have been shown to be able to create ion selective pores in polymersome membranes with thicknesses up to approximately 12 nm, but no greater [36,37]. Successful reconstitution of IMPs into polymer membranes >10 nm in thickness, resulting in a large hydrophobic mismatch with the protein, has also been readily achieved [30].

Several highly stable membrane proteins have been successfully reconstituted into polymer vesicles, with outer membrane protein F (OmpF), bacteriorhodopsin (BR) and AquaporinZ (AqpZ) being common examples. OmpF, for example, is known to be able to withstand harsh conditions, such as high temperatures, proteases and denaturing detergents [38,39]. A broader range of examples of membrane proteins reconstituted into polymersomes is given in Table 1. This list is not intended to be exhaustive; a more comprehensive record can be found in Table 1 of reference [40].

**Table 1:** Examples of IMPs reconstituted into polymersomes

Protein	Polymer(s)	Notes	Ref.
OmpF	PBd_12_-PEO_8_	Magnetic fields can be used to drive OmpF crystallisation.	[58]
	PMOXA_6_-PDMS_44_-PMOXA_6_	OmpF gated with a pH-responsive cap.	[59]
BR/F-ATPase	PEtOz_11_-PDMS_76_-PEtOz_11_	Co-reconstitution of BR and F-ATPase allows coupling of membrane protein function: light-driven ATP synthesis. Polymer membranes support pH gradients sufficient to create a proton-motive force to drive secondary IMP functions. Choice of reconstitution method flipped the preferred orientation of BR in vesicles allowing selection of vectorial proton transport into or out of the vesicle.	[46,47]
PR	P4MVP*_x_*-PS*_y_*-P4MVP*_x_* (*x*,*y*) = (21.26), (21.38), or (29.42)	Reconstitution into highly stable glassy membranes. Electrostatically driven protein reconstitution. Membrane acts as an allosteric regulator of PR function.	[43]
AqpZ	PMOXA_15_-PDMS_110_-PMOXA_15_	Polymer membranes alone are impermeable to water. AqpZ allows water to cross the membrane but not larger solutes.	[60]
Aqp0	PMOXA_20_-PDMS_75_-PMOXA_20_ PEO_25_-PDMS_40_-PMOXA_110_ PEO_67_-PDMS_40_-PMOXA_45_	Investigates oriented insertion into symmetric ABA and asymmetric ABC membranes. Relative sizes of PEO and PMOXA hydrophilic blocks determine polymer orientation in the vesicle membrane: the larger block generates positive curvature and forms the outer membrane surface. Preferential protein orientation only observed for ABC polymer vesicles.	[31]
	PBd_10_-PEO_12_ PBd_22_-PEO_14_ PMOXA_20_-PDMS_42_-PMOXA_20_ PMOXA_12_-PDMS_55_-PMOXA_12_	High densities of Aqp0 can be functionally reconstituted into polymersomes. Effects on vesicle morphology observed for high protein concentrations.	[61]
FhuA	PIB_18_-PEO_136_-PIB_18_	The protein is re-engineered to increase the hydrophobic β-barrel length by 1 nm to allow for more favourable solvation interactions with the membrane.	[51]
KcsA	PMOXA*_x_*-PDMS*_y_*-PMOXA*_x_* (*x*,*y*) = (6.34), (7.49), or (12.63)	Flexibility of PDMS block allows insertion into membranes with large hydrophobic mismatch. Even with a large hydrophobic mismatch, the fluidity of the PDMS chains means that the protein diffusion constant is only one order of magnitude slower than in a lipid bilayer. No evidence for functional incorporation of KcsA in these membranes.	[30]
Integrin α_v_β_3_	PBd*_x_*-PEO*_y_* (*x*,*y*) = (22.14), (17.6), or (12.9)	*In vitro* (cell free) membrane-assisted protein synthesis. Integrin incorporation efficiency is not found to be dependent on the polymer block length.	[44]
Complex I	PMOXA*_x_*-PDMS*_y_*-PMOXA*_x_* 8 polymers 9 ≤ *x* ≤ 65 23 ≤ *y* ≤ 165	Transmembrane electron transfer from NADH to an encapsulated quinone. Increasing membrane thickness increases the activity of complex I. Increasing hydrophilic polymer length at fixed hydrophobic thickness decreases complex I activity. Specific inhibition by 10 μM piericidin A reduces activity by >90%.	[42]
LamB	PMOXA_11_-PDMS_73_-PMOXA_11_	LamB acts as a specific receptor for λ phage to trigger DNA loading into the polymersome lumen.	[62]
TsX	PMOXA_20_-PDMS_54_-PMOXA_20_	200 nm polymersomes with the nucleoside-specific porin TsX. Encapsulation of thymidine phosphorylase for enzyme-replacement therapy for mitochondrial neurogastrointestinal encephalomyopathy. Nanoreactors are functional in serum at 37°C, show low cytotoxicity and do not stimulate a significant inflammatory response.	[63]
Claudin-2	PBd_21_-PEO_12_	Cell-free protein expression and directed insertion into polymersomes. Protein in polymersomes confirmed by specific antibody binding (SPR).	[64]
GPCR (5-HT_1A_R)	PBd_12_-PEO_9_	Functional reconstitution into giant polymer vesicles. Oriented protein insertion with ∼90% of GPCR in its native orientation. GPCR activity retained after lyophilisation and rehydration of vesicles.	[50]

Reconstitution methods into polymersomes bear similarities to those used for proteoliposomes. For example, polymersomes can be formed from lipid-detergent micelles [41], temporary destabilisation of preformed polymersomes detergents [42] and spontaneous insertion of the protein into the membrane [43]. Detergent removal can be achieved by the use of BioBeads or dialysis. Removing detergent by diluting them to below the critical micelle concentration (CMC) and harvesting the polymersomes by centrifugation may not be possible as, unlike liposomes, many polymersomes are less dense than water and so cannot be spun down into a pellet. Direct incorporation of membrane proteins into polymersomes from cell-free synthesis has also been reported [44]. However, differences in properties between lipid and polymer systems mean that, for a given IMP, successful proteoliposome reconstitution protocols are not necessarily directly transferable to polymersome systems. Unlike lipid–detergent interactions [11,45], detergent–copolymer interactions are currently not as well understood, making rational modification of IMP–polymersome reconstitution protocols a major challenge.

Several studies show the potential of polymersomes as platforms for a wider range of advanced IMP systems. Different IMPs have been functionally reconstituted into the same polymersome, most elegantly demonstrated by the coupling of BR and *F*_0_−*F*_1_ ATP synthase (F-ATPase) for light-generated ATP synthesis [46,47]. Reconstitution of more challenging IMPs into polymersomes may also be possible through careful optimisation of polymer structure, chemistry and reconstitution protocols. For example, complex I of the electron transport chain has been functionally reconstituted into PMOXA–PDMS–PMOXA triblock copolymer vesicles [42].

Polymer membranes might also offer some functional advantages over traditional proteoliposomes. Oriented reconstitution of an Aquaporin0 (Aqp0) channel has been achieved using asymmetric ABC PEO–PDMS–PMOXA triblock membranes (Figure 1), opening the possibility, for example, of directional substrate transport into/out of vesicles containing multiple reconstituted IMPs [31]. While natural biomembranes have an asymmetric lipid distribution, asymmetric lipid bilayer vesicles are much more challenging to fabricate for oriented membrane protein reconstitution [48,49]. Furthermore, while conventional thinking says that block copolymers for IMP reconstitution should be in a fluid state, above their glass transition temperature, proteorhodopsin (PR) has been functionally reconstituted into highly stable glassy block copolymer membranes with PS hydrophobic blocks [43]. Proteopolymersomes have also shown stability under lyophilisation: following rehydration, PBd–PEO vesicles are restored without a significant loss of IMP function, in this case a G-protein-coupled receptor [50].

Polymersomes are not always favourable environments for IMP reconstitution and modifications to the protein or membrane environment may be required to achieve the desired function. Evolution has optimised membrane proteins for function within lipid membrane matrices; however, protein engineering might be used to adapt a membrane protein for more favourable insertion into synthetic polymer membranes. The ferric hydroxamate protein uptake component A (FhuA) has been engineered to increase its hydrophobic surface by 1 nm, reducing hydrophobic mismatch and lowering the insertion penalty into thicker polymer membranes [51]. However, hydrophobic mismatch is not the only challenge; many membrane proteins require specific interactions with lipids for native function [52,53]: for example, recent reports of specific lipid regulation of the TrpV1 ion channel [54]. Even if specific lipid interactions are not required, the delicate balance of forces experienced by an IMP in a biomembrane may be important for maintaining its native structure and/or function, i.e. establishing a lateral pressure profile in the membrane that mimics the natural biomembrane [55–57]. These issues motivate the modification of polymersome properties to enhance their biofunctionality. This has been approached by blending block copolymers and phospholipids to create hybrid vesicles with the goal of combining the best features of these two materials: the chemical versatility and robustness of polymersomes with the biocompatibility and biofunctionality of liposomes.

## Hybrid vesicles

**Figure 2.**
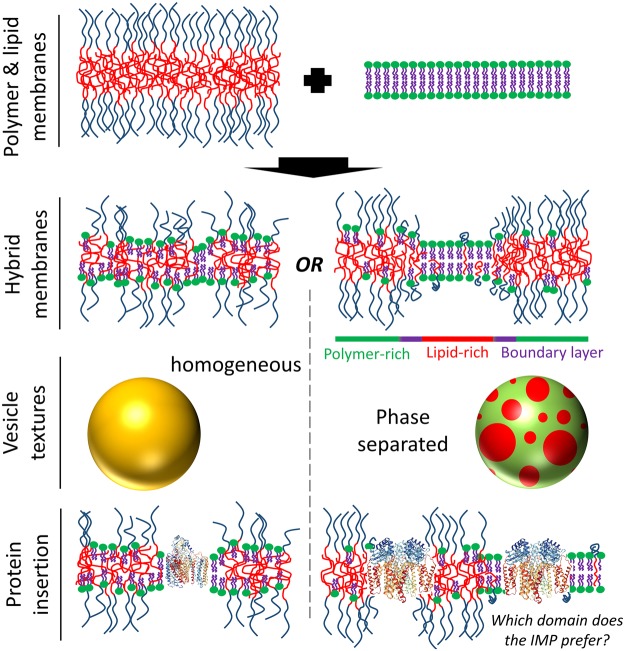
Hybrid vesicles. Hybrid vesicles combine lipids and block copolymers into blended membranes. These hybrid membranes can either be well mixed, giving homogeneous properties across the surface of the vesicle (left-hand side), or phase separated into lipid-rich and polymer-rich domains, which give rise to textured vesicle morphologies with coexisting domains of different structures and properties (right-hand side). Membrane proteins can be inserted into these hybrid membranes, either into homogeneous membranes (left-hand side, showing cyt bo_3_; PDB ID: 1FFT) or phase-separated membranes, where the preferred location of the IMP in the membrane is dependent on the relative properties of these two coexisting phases (right-hand side, showing MloK1; PDB ID: 4CHW).

Given the disparity in properties between lipid and block copolymer membranes, in particular differences in membrane thickness and structure, it may seem surprising that blends of lipids and block copolymers can form mixed, hybrid vesicles (Figure 2). Therefore, the study of the mixing behaviour between lipids and polymers in hybrid vesicles has been at the fore of their material characterisation.

Several block copolymer classes have been investigated in vesicle blends with phospholipids; examples include PBd-PEO [65–67], PDMS-PMOXA [68], PIB-PEO [69], PChA-PNIPAAm [70] and PDMS-PEO [71,72]. These mixtures can form homogeneous, well-mixed membranes or phase separate into lipid-rich and polymer-rich domains dependent on several factors, including lipid polymorphism and phase transitions, hydrophobic mismatch, cross-linking between lipids or polymers and specific mixing interactions between the individual components. While well-mixed vesicles [65] may give optimal, modified local membrane environments for protein reconstitution, lipid-rich domains within a bulk polymer matrix may also be desirable for tuning optimal activity and durability. In particular, nanodomains of lipids have been reported in PDMS-PEO/POPC hybrid vesicles [73], where the PDMS hydrophobic block is less compatible with the lipid tail groups than a PBd polymer block based upon their respective solubility parameters [74]. Stable, nanoscale lipid-rich islands within a sea of mechanically robust polymer-rich membrane might be particularly attractive IMP-functionalised constructs for combining native-like lipid solvation with the enhanced structural stability of polymersomes.

Hybrid vesicles tune the dynamical and mechanical properties of vesicle membranes between those of pure lipids and pure polymers [66,72,75]. Lipid-specific stimuli can also be used to address the hybrid membranes, particularly when phase-separated into lipid-rich domains, modifying structure and permeability [67]. We point the reader to more comprehensive reviews of the physicochemical properties of hybrid vesicles for further details on this topic [74,76].

The study of membrane proteins in hybrid lipid–polymer systems is still in its infancy with only one report currently studying vesicle architectures. A summary of current reports is presented in Table 2. Phase separation and mixing phenomena are important properties in hybrid materials, as we discuss above. This begs the question as to where a membrane protein would prefer to reside in textured, phase-separated hybrid membranes. The partitioning of OmpF in lipid–polymer Langmuir monolayers at the air–water interface have shown that the protein can prefer to reside in polymer-rich domains [77]. At first sight this is a surprising result as the motivation for using hybrid membranes is the preference of membrane proteins for native lipid environments. However, the lipid-rich domains (DPPC) in the present study are in a non-native gel phase: the ordering and tight packing of the lipids in this phase exclude impurities, e.g. OmpF, causing them to instead reside in the more flexible and fluid polymer-rich regions of the membrane.

**Table 2:** Overview of membrane protein reconstitution into hybrid lipid–polymer systems

Protein	Polymer(s)	Lipid(s)	Membrane architecture	Notes	Ref.
OmpF	PMOXA_15_-PDMS_110_-PMOXA_15_	DPPC	Langmuir monolayers	Phase separation between lipid and polymer components creates textured films. Protein preferentially partitions with fluid polymer domains, excluded from the non-native lipid gel phase domains. Native structure or function of the reconstituted protein is not studied.	[77]
	PI*_x_*-PEO*_y_* (*x*,*y*) = (9.6), (16.10) or (30.26)	DPhPC	Planar membranes	Three different polymer lengths studied at a 90:10 ratio with lipid. Channel conductance comparable with that in pure lipid for hybrid membranes of all polymer lengths. Native-like voltage-dependent channel closing is observed. Hybrid membranes inhibit protein insertion compared with pure lipid. Comparison of hybrids with the pure polymer system is not made.	[79]
MloK1	PDMS*_x_*-PMOXA*_y_* (*x*,*y*) = (65.12), (37.9) or (16.9)	DPPC, DOPC, DPPE or POPE	Solid-supported planar membranes	Three different length polymers are studied in conjunction with one of four different lipids. Protein insertion into phase-separated lipid–polymer membranes shows that the protein partitions based on the fluidity of coexisting domains, disfavouring lipid gel phases and favouring fluid lipid domains. Native structure or function of the reconstituted protein is not studied.	[78]
Cyt *bo_3_*	PBd_22_-PEO_14_	POPC	Vesicles	Compositions from 0 to 100% polymer content in 25% increments. Only a small drop in protein activity is observed for up to 50% polymer; activity drops significantly above 50% polymer content. The functional lifetime of the protein is significantly extended with increasing polymer content. Evidence that purification of vesicles from coexisting micelles could further enhance the durability of function.	[80]

Similarly, the observation that membrane proteins (MloK1) prefer polymer-rich membrane over solid-like gel phase lipid (DPPC, DPPE) was further demonstrated in planar, solid-supported membranes (Figure 2) [78]. Importantly, in separated PDMS-PMOXA/DOPC membranes, where DOPC-rich domains were observed, MloK1 preferentially partitions into the fluid lipid-rich phase. Furthermore, PDMS-PMOXA/POPE membranes form well-mixed membranes with uniform protein distribution. This reveals an important control parameter: the location of protein within a membrane can be manipulated between polymer-rich or lipid-rich domains or a uniform membrane distribution by judicious choice of the lipid.

Crucially, these two aforementioned studies do not investigate native structure or function of the membrane protein reconstituted within hybrid membranes. This shortfall has been addressed for OmpF in PI-PEO membranes (90:10 polymer:protein) [79]. Electrochemical analysis of the protein conductivity shows comparable values between lipid (DPhPC) and polymer-rich (PI-PEO/DPhPC) membranes. This result was independent of the length of polymer used. OmpF also exhibited native-like voltage-dependent channel closing. However, protein insertion into polymer-rich membranes was suppressed in hybrid membranes compared with pure lipids. The authors suggest that this may be due to excess residual chloroform in hybrid films that denature the protein. Notably, there is no comparison of protein properties within 100% polymer membranes that convincingly justifies the requirement of a more complex hybrid membrane; OmpF has been functionally reconstituted into polymersomes as earlier discussed.

The advantage of the hybrid system became evident for cytochrome *bo_3_* (cyt *bo_3_*) reconstituted into PBd-PEO/POPC vesicles [80]. Here, the protein is not functional in the pure polymersome system. However, there is only minimal loss in protein activity for vesicles with up to 50% polymer content. Impressively, increased polymer content enhances the functional lifetime of the protein, with 50% polymer hybrids providing the best combination of high initial activity and durability of function. Intriguingly, purification of these 50% polymer proteo-hybrid vesicles by size exclusion chromatography to remove coexisting micelles suggests a further improvement in cyt *bo_3_* durability with a ∼20% drop in activity after one week followed by a very slow decline in function up to week 6 where >70% of its initial activity still remains. As a comparison, control proteoliposomes rapidly lost function and were inactive within four weeks. In future it will be of interest to conduct a more long-term study of the activity of these hybrid vesicles, beyond 6 weeks, to understand the full extent of their extraordinary stability.

## Outlook

Stability and durability are important characteristics for membrane proteins to make a successful impact in biotechnologies. Where compartmentalisation via vesicular architectures is required, proteoliposomes are unlikely to meet these obligations. More robust block copolymer membrane systems will be important in this provision but, beyond the most stable and robust membrane proteins, the non-native polymersome environment will limit the inventory of viable protein components. Protein engineering to optimise the protein's structure for incorporation within the polymer membrane may yield some success, e.g. by matching their hydrophobic thickness, but optimisation of the membrane matrix for biofunctionality is likely to be a more straightforward and fruitful approach with broad applicability to a wide range of membrane proteins. Blending lipids and polymers within a membrane environment will allow native-like lipid solvation of the protein and facilitate specific lipid–protein interactions that may be important for function and to tune the local fluidity and mechanics of the membrane environment that the protein experiences.

Investigation of hybrid vesicles for membrane protein reconstitution is still at an immature stage of development. Much work needs to be done to further optimise the hybrid membrane environment, which will be best achieved through a more detailed fundamental understanding of the coupling between the membrane composition and its bulk physicochemical properties. Furthermore, a much wider range of membrane proteins needs to be investigated when reconstituted into hybrid vesicles to test the generality of this approach. This selection of proteins must extend beyond the most stable model IMPs into important IMPs that are more difficult to handle in order to fully challenge the abstraction of this principle. However, there is nothing unique about cyt *bo_3_* that would suggest that its enhanced durability in hybrid vesicles is a special case [80]. This enhanced stability may not only be critical to synthetic biology but could also become an important tool in handling membrane proteins in fundamental biochemical studies where protein stability has proved to be a major impediment. A more long-term appeal for these systems is not just to create a stable, functional environment, but to use techniques such as lipidomics to accurately reflect the lipids found within the native environment, as has been done with the SMALP platform [81]. Rapid recent advances in vesicle engineering are beginning to overcome the practical challenges in realising robust biotechnologies based on these hollow capsules. This augurs a bright future for emerging applications, encompassing nanoreactor, drug delivery and biosensor technologies, among others.

## Abbreviations

Aqp0: Aquaporin0
AqpZ: AquaporinZ
BR: Bacteriorhodopsin
Cyt *bo_3_*: Cytochrome *bo_3_* ubiquinol oxidase
DOPC: 1,2-dioleoyl-sn-glycero-3-phosphocholine
DPhPC: 1,2-diphytanoyl-sn-glycero-3-phosphocholine
DPPC: 1,2-dipalmitoyl-sn-glycero-3-phosphocholine
DPPE: 1,2-dipalmitoyl-sn-glycero-3-phosphoethanolamine
F-ATPase: F_0_F_1_ ATP Synthase
FhuA: Ferric-hydroxamate uptake protein A
GPCR: G-protein-coupled receptor
IMP: Integral membrane protein
KcsA: Potassium crystallographically sited activation channel
LamB: Bacteriophage lambda receptor (maltoporin)
MloK1: Cyclic nucleotide-modulated potassium channel 1
OmpF: Outer membrane protein F
P4MVP: Poly(4-vinyl-*N*-methylpyridine iodide)
PBd: Polybutadiene
PChA: Poly(cholesteryl acrylate)
PDMS: Poly(dimethyl siloxane)
PEO: Poly(ethylene oxide)
PEtOz: Poly(2-ethyl-2-oxazoline
PI: Poly(1,4 isoprene)
PIB: Polyisobutylene
PMOXA: Poly(2-methyl oxazoline)
PNIPAAm: Poly(N-isopropylacrylamide)
POPC: 1-palmitoyl-2-oleoyl-sn-glycero-3-phosphocholine
POPE: 1-palmitoyl-2-oleoyl-sn-glycero-3-phosphoethanolamine
PR: Proteorhodopsin
PS: Polystyrene
SPR: Surface Plasmon Resonance
TsX: Nucleoside-specific channel forming protein TsX
